# Predicting the distributions of Egypt's medicinal plants and their potential shifts under future climate change

**DOI:** 10.1371/journal.pone.0187714

**Published:** 2017-11-14

**Authors:** Emad Kaky, Francis Gilbert

**Affiliations:** 1 School of Life Sciences, University of Nottingham, Nottingham, United Kingdom; 2 Kalar Technical Institute, Sulaimani Polytechnic University, Sulaymaniyah, Iraq; University of Sydney, AUSTRALIA

## Abstract

Climate change is one of the most difficult of challenges to conserving biodiversity, especially for countries with few data on the distributions of their taxa. Species distribution modelling is a modern approach to the assessment of the potential effects of climate change on biodiversity, with the great advantage of being robust to small amounts of data. Taking advantage of a recently validated dataset, we use the medicinal plants of Egypt to identify hotspots of diversity now and in the future by predicting the effect of climate change on the pattern of species richness using species distribution modelling. Then we assess how Egypt's current Protected Area network is likely to perform in protecting plants under climate change. The patterns of species richness show that in most cases the A2a ‘business as usual’ scenario was more harmful than the B2a ‘moderate mitigation’ scenario. Predicted species richness inside Protected Areas was higher than outside under all scenarios, indicating that Egypt’s PAs are well placed to help conserve medicinal plants.

## Introduction

Climate is one of the main elements used to describe plant niches, and climate change is considered one of the major threats to biodiversity [1–4]. One of the main challenges today is to forecast how the various climate-change scenarios might affect species and communities in the future [2, 5–8]. To reduce the effects of climate change on biodiversity, we should be taking precautionary measures, in the long term to reduce emission gases, and in the short term to design appropriate networks of Protected Areas (PAs) for conservation [9].

Here we apply species distribution models (SDMs) to predict the impact of climate change on shifting distributions of Egyptian medicinal plants, and to evaluate the effectiveness of the Egyptian network of reserves. Medicinal plants are the main source of new herbal products and medicines [10–12], and therefore they are important for their roles in human health, the economy especially of poor areas, and culture and heritage [13, 14]. Between 70 and 80% of people across the world use medicinal plants as their traditional and primary system of health care [15, 16]. Because of the growing demand for natural health products and herbal medicine, the use of medicinal plants is increasing [10]; in 1999 their annual market value was evaluated at $US 20–40 billion, with an annual growth rate of 10–20% [17]. About 50000–80000 plant species are used for medicinal purposes across the world [10], with very variable distribution [18, 19] and mostly harvested from the wild [20]. Their current extinction rate is 100 to 1000 times higher than the natural background extinction rate, implying the loss of at least one important drug every two years [10, 21]. It is therefore very important to conserve these kinds of plants, by investigating their ecological requirements and increasing the awareness of decision makers, stakeholders and public [22].

SDMs create a model of the relationship between species distribution and environmental predictors (such as climate, land cover/use, and habitat), which can then be projected under different climate scenarios [4, 23, 24]. Modelling techniques rather than field data are more effective in evaluating the spatial reaction to climate drivers over large spatial and temporal scales [6]. SDM methodology has developed very quickly so that currently there are many algorithms, platforms and software available. In our case study of the data-sparse country of Egypt, we have tried to keep the modelling as simple as possible, and hence we use just one SDM method (MaxEnt) with proven abilities in this field [25, 26], especially in its ability to produce reasonable models with sparse data [27, 28]. The majority of countries find themselves in the position of having to make decisions about conservation in the face of climate change, but with few data to guide them.

SDMs are also used to assess the likely future value of PAs, important fundamental units in conservation which cover 14.8% of the entire land area of the world [29]. There is a big effort to enlarge the coverage of PAs to 17% by 2020 [30]. The important question is to what extent these PAs can preserve species under climate change, recognising that many species will shift their distributions. Current PAs might still achieve significant roles if they provide suitable areas for species to disperse into new districts [31]. The best strategy to preserve biodiversity is to recognise and protect the best locations [32]—but are PAs in the “best” locations? There are several studies that measure current species richness inside and outside PAs; some found that species richness is higher inside [22, 28, 33], while others found the opposite [34, 35]. In this case study, we use species richness as our criterion to show the effectiveness of Egypt's network of PAs, given that the preservation of 'biodiversity' is their objective in nearly all cases.

We focussed on medicinal plants because (a) a validated dataset exists, unlike for all plants; and (b) they are subjected to unregulated harvesting within Egypt by pharmaceutical companies, and hence there is more concern about them than other plants. Thus we evaluated the pattern of species richness for 114 Egyptian medicinal plants projected into the future to ask what the predicted patterns of diversity, gains, losses and turnover are under the various climate-change and dispersal scenarios. Then we ask whether Egypt’s PAs provide and will provide suitable plant habitat compared with outside the PAs. There are about 30 PAs in Egypt covering approximately 15% of Egypt’s land area [36]. PAs are imperfect in their coverage of biodiversity [37], but because of their extent Egyptian PAs at least in theory protect biodiversity much better than those of many other countries [28]. In all this work, “species richness” is understood to mean a quantitative estimate of the collective habitat suitability of all the 114 species combined, since that is what the raw MaxEnt output actually means.

## Materials and methods

Egypt has a total of 2174 recorded species [38]. Botanists have judged 121 of them as known to be ‘medicinal, but these are not representative of all Egypt’s plants, with certain families (e.g. Chenopodiaceae, Labiatae, Zygophyllaceae) greatly over-represented, and some (e.g. Graminae, Leguminosae-Papilionoideae) greatly under-represented. The data were collated by the BioMAP project in Cairo between 2004–2008, funded by Italian Debt Swap [33]; the data are presence-only, collected from different sources (i.e. literature, herbarium, and field work: see Supplementary S6 Table), but mainly recent fieldwork especially in Sinai. There are virtually no available validated records of these species from surrounding countries, so we were unable to model the entire range of non-endemic species; this will often be the case for developing countries. All the data were examined in detail by a panel of expert field botanists during the BioMAP project, filtering out all records that were dubious on a variety of grounds (mainly identification and distribution). Much of the data derive from recent (since 1990) expeditions of academic botanists using GPS recorders, particularly to the wadis of Sinai, where plots were surveyed at approximately regular distances apart. Earlier records were carefully checked for the accuracy of the locations, and records with vague data removed. We removed species with fewer than ten spatially separate records, and those where the records were spatially very close together and with mean AUC of the models less than 0.5 (all to avoid overfitting [26]). We eventually used 114 species consisting of 14396 point records (S5 Table).

The environmental variables were 23 predictors, 19 of them (bioclimatic variables) downloaded from the WorldClim v1.4 dataset at a resolution of 2.5 arc-minutes (http://www.worldclim.org/bioclim) [39] (see Table 1). The Normalized Difference Vegetation Index (NDVI) data for seven years (2004 to 2010) were downloaded from the SPOT Vegetation website (http://free.vgt.vito.be/). These data are made available as maps synthesized over 10-day periods at resolution of 1 km. 252 maps represent data from 2004–2010, which were then clipped to Egypt's boundaries and used to create two predictors—maximum (Max_NDVI, indicating the maximum amount of vegetation there is per pixel) and the difference between the Minimum and Maximum (NDVI_differences, indicating the variability in vegetation per pixel). The maps were then rescaled to 2.5 arc minutes [36]. The predictor layer of ‘habitat’ was created by BioMAP [28], dividing Egypt into eleven classes (sea, littoral coast, cultivation, sand dune, wadi, metamorphic rock, igneous rock, gravels, serir sand sheets, sabkhas and sedimentary rocks). Altitude data were downloaded and rescaled to a pixel size of 2.5 arc-minutes [36].

**Table 1:** Environmental variables used to build the models (highlighted); non-highlighted variables were removed after applying the Variance Inflation Factor (VIF) analysis to reduce collinearity.

BIO1	Annual Mean Temperature
BIO2	Mean Diurnal Range (Mean of monthly (max temp—min temp))
BIO3	Isothermality (BIO2/BIO7) (* 100)
BIO4	Temperature Seasonality (standard deviation *100)
BIO5	Max Temperature of Warmest Month
BIO6	Min Temperature of Coldest Month
BIO7	Temperature Annual Range (BIO5-BIO6)
BIO8	Mean Temperature of Wettest Quarter
BIO9	Mean Temperature of Driest Quarter
BIO10	Mean Temperature of Warmest Quarter
BIO11	Mean Temperature of Coldest Quarter
BIO12	Annual Precipitation
BIO13	Precipitation of Wettest Month
BIO14	Precipitation of Driest Month
BIO15	Precipitation Seasonality (Coefficient of Variation)
BIO16	Precipitation of Wettest Quarter
BIO17	Precipitation of Driest Quarter
BIO18	Precipitation of Warmest Quarter
BIO19	Precipitation of Coldest Quarter
Altitude	Altitude
Habitat	Habitat
NDVI_Max	NDVI maximum value
NDVI_Difference	Absolute difference between the highest and lowest NDVI values

Eleven of these 23 predictors (Table 1) were eventually used after removing collinearity by applying the Variance Inflation Factor using R v2.15 (the *'car'* package: R Development Core Team 2012). The predictors with the maximum VIF values were discarded first, and new VIF scores calculated among the remaining predictors; this was repeated until all VIF scores were under 10.

Models for the current distribution of each species were projected into the future at the standard three different time slices (2020, 2050, and 2080) using predicted future climates from the IPCC’s 4^th^ assessment [40] taken from the International Centre for Tropical Agriculture website (see http://www.ccafs-climate.org/), selecting two HadCM3 scenarios (A2a and B2a). Such Global Circulation Models are widely used in SDMs to explore the effect of climate change on biodiversity [5, 41, 42], including likely shifts in distribution, habitat change, gains and losses, turnover and extinction [43, 44]. We chose to use the IPCC 4th assessment, rather than the latest 5th assessment and its very different scenarios, for continuity with previous work (e.g. [36]) and because the differences in SDMs are slight [45].

The A2 and B2 scenarios [40] are regularly used in climate-change assessments [1]. Both scenarios have different assumptions about the levels of CO_2_ emissions. The A2 scenario expects this level to increase without barriers because of high growth rate in human population, not much technological development, expanded land-use change, and less environmental awareness. The B2 scenario expects the level not to change much because human population growth will be slower, with fewer changes in land-use, people are more environmentally conscious, and there is increasing technological invention [46, 47]. In this study, we do not take into account any phenological and/or evolutionary reactions to climate change, and hence we assume that species will attempt to find their climatically suitable habitat dependent on their dispersal capability. We are forced to make the assumption that some predictors (e.g. habitat, NDVI) do not change because there is no information or scenarios on how they might change; others (e.g. altitude) will clearly not change.

Maximum Entropy (MaxEnt) version 3.3.3k [48] was used to run the models, choosing the options that created the best models (i.e. feature classes QPT, 10000 background points, 1000 iterations, cross-validation [k = 10] with 10 replicates [for estimating prediction errors], 10% training presence threshold, and logistic output format), and to create the binarized (via thresholding) maps. MaxEnt performance is good with presence-only data and relatively few records [24, 49]. The option set was chosen after extensive tests to maximise the AUC and TSS, the standard measures of goodness-of-fit for SDMs [50]. The one species with a mean AUC score less than 0.7 was removed from the analysis, as recommended [51]. Following [52, 53], we chose the “10% training presence” threshold rule to give a binary map for each of the 10 replicates for each species. With the 10% training presence rule, the threshold selects pixels where 90% of the training presences are correctly classified, thus giving some allowance for the possibilities of ephemeral populations or recording errors. A minimal training presence threshold that correctly predicts every training presence may lead to over-prediction. The resulting maps were analysed under the two extreme dispersal assumptions (unlimited and no-dispersal) usually applied in such studies (for example, [43, 44, 54]). Both assumptions have been criticized for not involving the impact of biotic interactions on species distributions [55], but there is no practical way of incorporating any putative interactions for Egyptian species. Species gains occur when species occupy new areas, while losses can occur when the habitat suitability is reduced for that area in the future. Species turnover is the differences between the current and future species composition of the same location, used as an indicator at large or regional scales [5].

MaxEnt does not automatically produce a consensus binary (presence/ absence) map from the 10 replicate runs, and hence this was made manually using the Raster Calculator and Reclassify tools in ArcGIS 10.2.2 (ESRI, USA). For unlimited dispersal, the consensus map allotted a ‘presence’ to a pixel that had presence values in more than 50% of the model runs (i.e. >5 replicates). Under the no-dispersal assumption, the consensus maps for the two time periods (e.g. ‘current’ and ‘2020’) were compared so as to allocate a pixel to be a ‘presence’ in the event that it was a ‘presence’ in both maps.

Maps for the distribution of species richness for current and future times were produced by summing all predicted consensus distribution maps for the individual species together (assuming either unlimited or no-dispersal in the future). Thus, maps were produced for species richness for future times (2020, 2050, and 2080) under both scenarios (A2 and B2) for both unlimited and no-dispersal assumptions, a total of 12 species-richness maps. Change maps were produced to aid interpretation by subtracting one map from another.

We then compared the species richness inside and outside PAs to see to what extent they protect Egyptian plants now, and in the future, using the oldest and largest 25 PAs, excluding just the most recent and smallest. We chose 2000 pixels at random, and then created a 50-km buffer around each PA. We chose 50 km as the tradeoff between a small value (to try to ensure the habitat outside is as similar as possible) and a figure large enough to ensure enough of the random pixels lay within it. The mean species richness was then calculated for the random pixels lying within each PA, and lying outside but within the appropriate buffer zone, creating paired values inside and outside of each PA. In the case of very small PAs which are smaller than one pixel (whose side is 4.64 km), species richness was taken to be the value for the pixel containing the PA. The paired difference inside-outside was calculated for each PA, and the mean difference tested in a one-sample t-test against the null hypothesis that the mean equals zero.

To estimate how much each species loses or gains in habitat suitability, the number of pixels gained and lost for each species was calculated (see S1 Fig for an example), and the gain maps (future times × scenarios emissions) were summed together across species to produce a “gain in suitability” map to demonstrate which locations are projected to gain more species in the future; sites can only gain species under the assumption of unlimited dispersal, and such sites may constitute potential new PAs in the future. A loss map shows locations predicted to lose suitability for species in the future (assuming either unlimited and no-dispersal).

Species turnover is defined as the number of species changes in specific locations [5, 43, 54], used as a suitable measure of change in species composition to evaluate the effect of climate change on biodiversity from regional to continental levels [5, 43, 54]. It is calculated as the differences between present-day and future species composition [5]. Locations with small turnover are predicted to remain suitable in the future, whereas locations with higher turnover indicate less stable habitat suitability with time. Species turnover for unlimited dispersal was calculated (following [5, 44, 54]) as SG + SL, where SG is the number of species gained, and SL is the number of species lost (see S1 Fig for details). The equation normally expresses turnover as a proportion of the species richness of each pixel, but this was unsuitable in the case of Egyptian plants because of the very low richness in areas of the Western desert, which made some of the proportional turnover values unreasonably large. Species turnover for no-dispersal is just equal to species losses because there can be no gains.

All mean values are given ± one SE. Plant nomenclature follows Boulos [38]. S11 Fig gives all the locations and areas names mentioned in the text.

## Results

**Fig 1:**
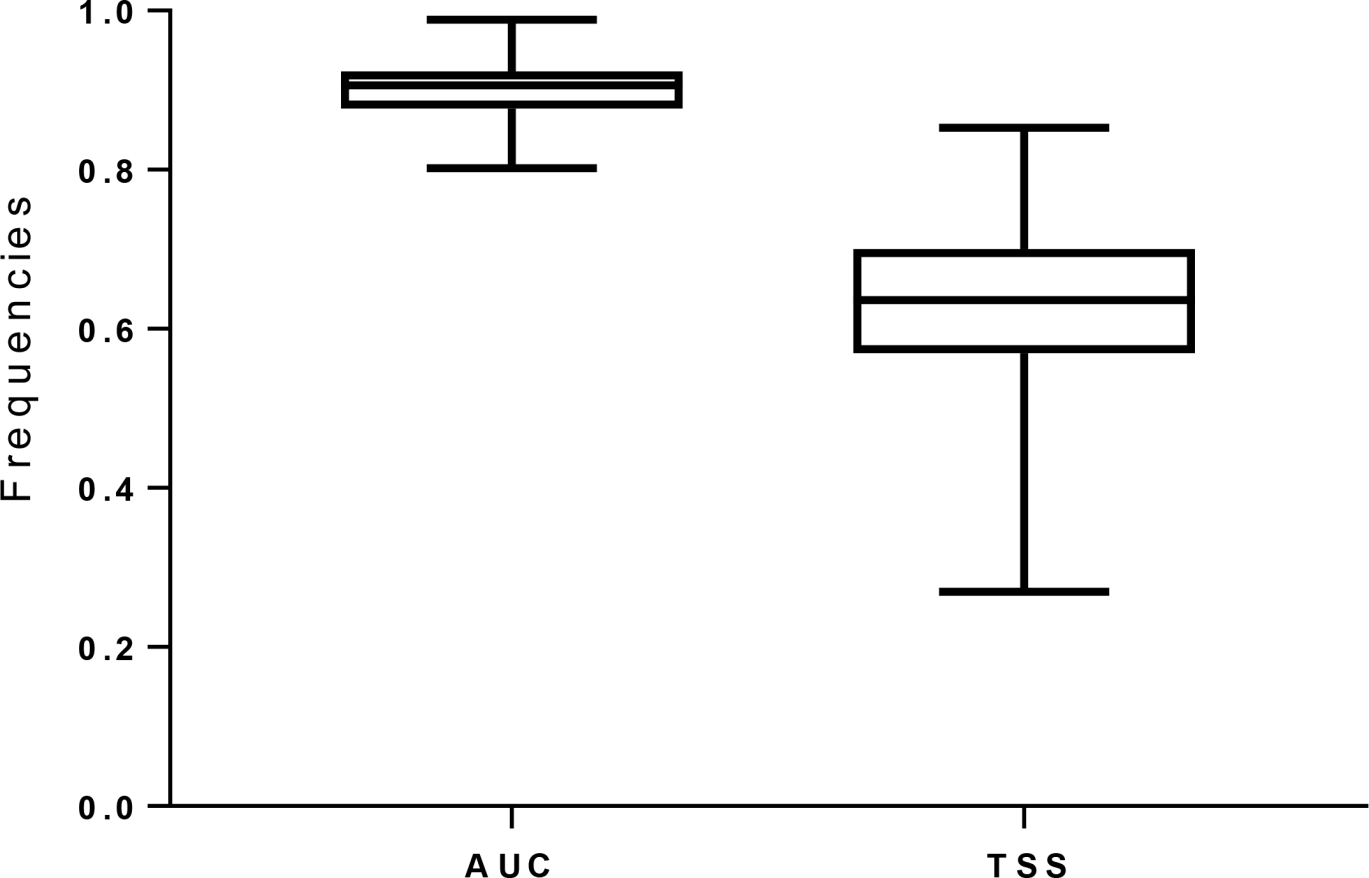
Boxplot of the Maxent predictive performance based on mean AUC and TSS scores.

The performance of the models was good in term of AUC (0.90 ± 0.004, 0.80 to 0.98) and TSS scores (0.63 ± 0.01, 0.27 to 0.85) (see Fig 1 and S5 Table); model performance was not affected by sample size. Three of the temperature-related variables had the most important effect on predicting distributions (see Table 1 and S12 Fig for details).

**Fig 2:**
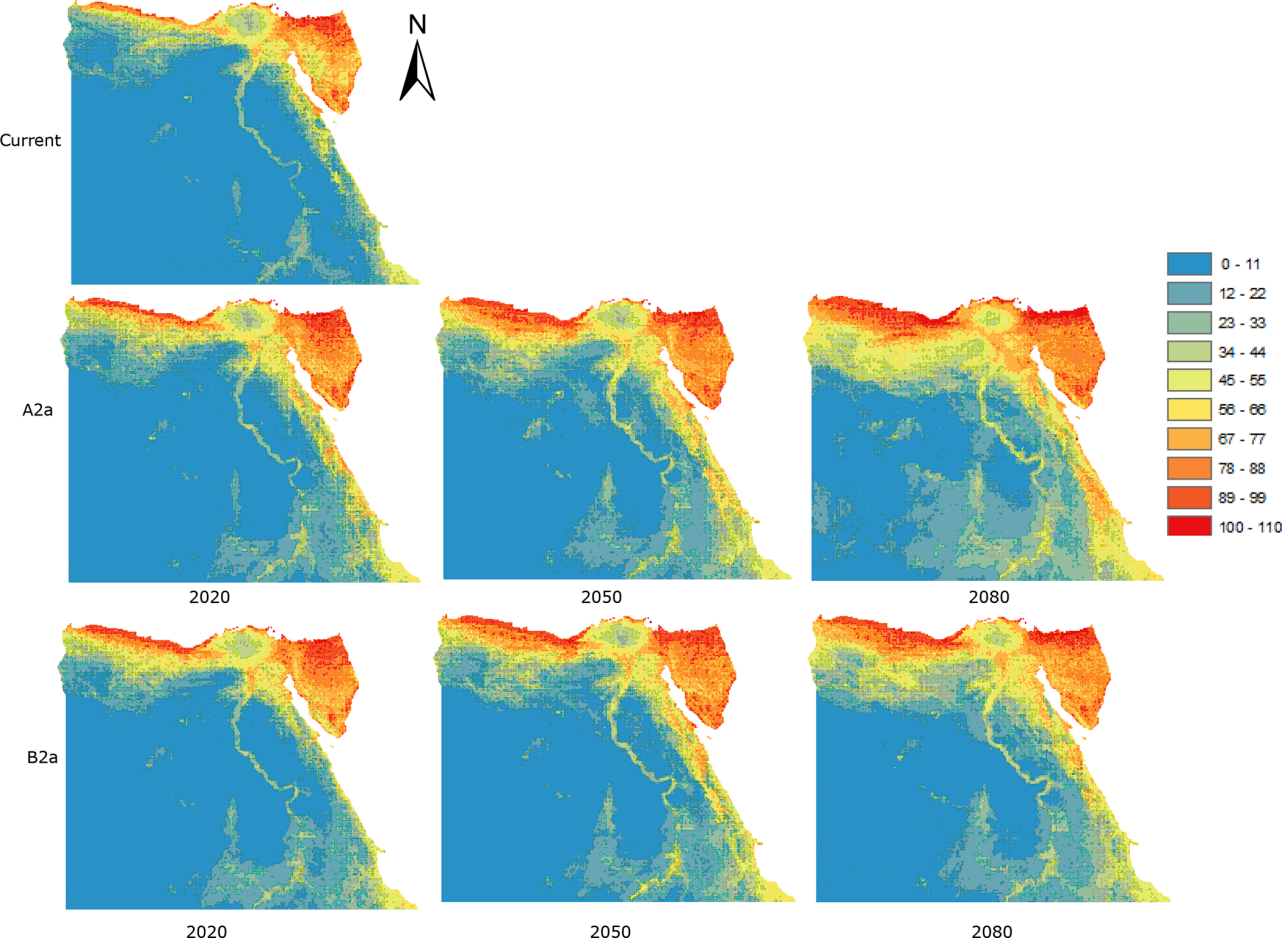
Predicted patterns of species richness using binary distributions for the present-day (‘current’) and into the future (2020, 2050, 2080) under two climate-change scenarios (A2a and B2a), created by adding together the maps of all 114 species and assuming unlimited dispersal. The colours indicate predicted species richness ranging between dark red (high) and blue (low).

**Fig 3:**
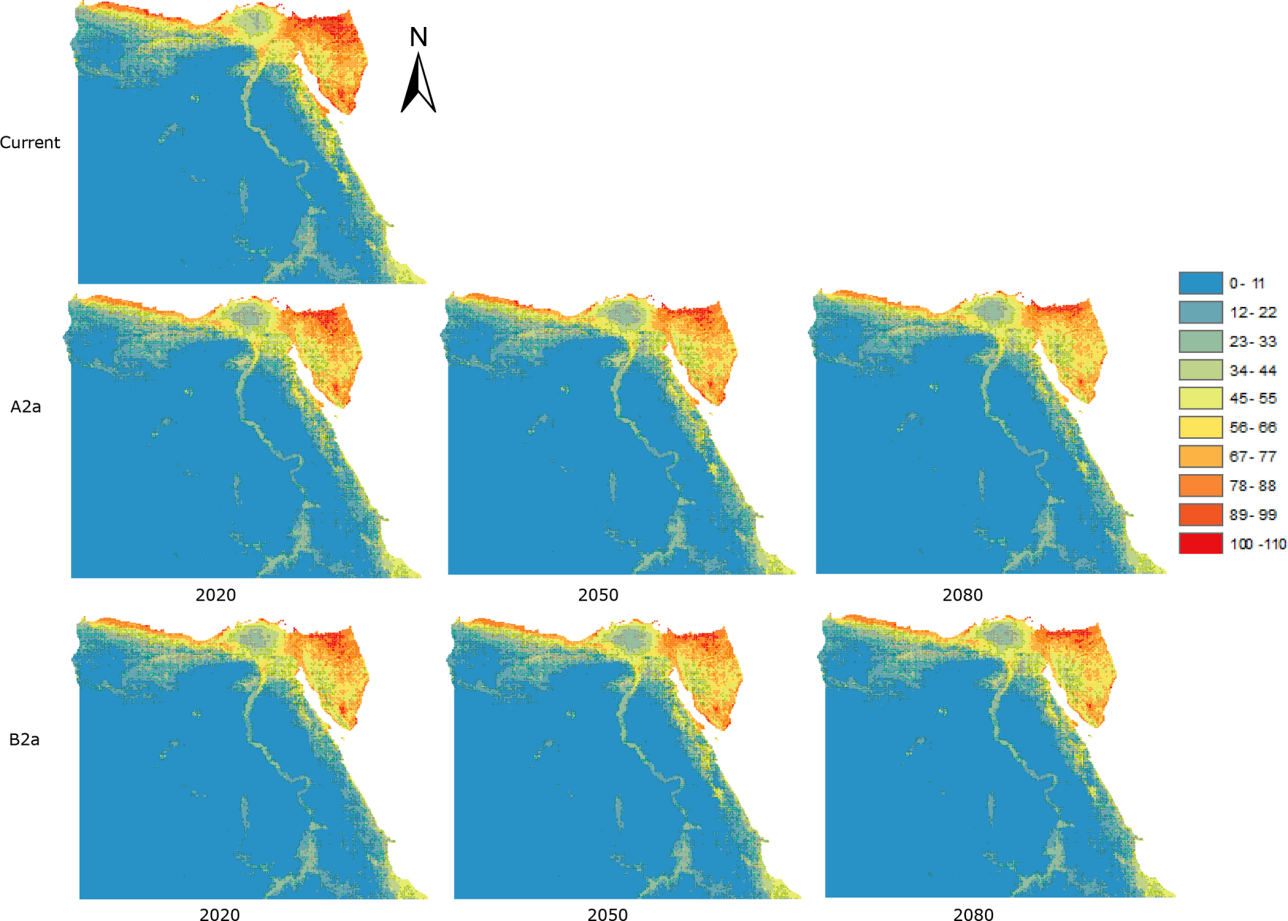
Data as in Fig 2, but assuming no-dispersal. The colours indicate predicted species richness ranging between dark red (high) and blue (low).

The pattern of species richness (Figs 2 and 3) increased toward the north and east of Egypt, especially the Mediterranean coast and Sinai. With unlimited dispersal, binary (Fig 2) species richness was predicted to increase for all future times (2020, 2050, and 2080) and both scenarios (A2 and B2). Using binary distributions, the pattern of species richness was predicted under both dispersal assumptions. For unlimited dispersal, by 2020 under the A2 scenario (Fig 2 and S2 Fig) species richness is predicted to increase along the Mediterranean coast and in the Qattara Depression, and in scattered areas on both sides of the Suez Gulf, the Red Sea coast, and Sinai. These predicted increases become more obvious by 2050 and very marked by 2080. Decreases in species richness are predicted to occur in small scattered areas, similar in all future time periods. There were clear differences under the B2 scenario (Fig 2 and S2 Fig); by 2020 the predicted increases are lower, and include some strong predicted declines along both sides of the Suez Gulf and further south along the Red Sea coast.

By 2050 species richness is predicted to increase generally, eliminating the previous declines. By 2080 the increases are predicted to be more marked in the same areas. Assuming no-dispersal, the predicted pattern under the A2 scenario (Fig 3 and S3 Fig) shows no change or slight declines by 2020, more marked on both sides of Suez Gulf, along the Red Sea coast, and some scattered areas around greater Cairo. By 2050, these predicted declines are less except for areas around greater Cairo. By 2080, predicted species richness declines are only marked on both sides of the Suez Gulf, the Red Sea coast, and in central Sinai and greater Cairo. Under the B2 scenario, the predicted pattern of decline is similar but the magnitudes are greater except for 2080 (Fig 3 and S3 Fig).

The overall average predicted mean species richnesses for binary distributions, assuming unlimited dispersal (S4 Fig), gradually increase for future times (2020, 2050, and 2080) under both A2 and B2 scenarios compared to the present day, while assuming no-dispersal results in predicted declines for both scenarios compare with present day (S5 Fig).

**Fig 4:**
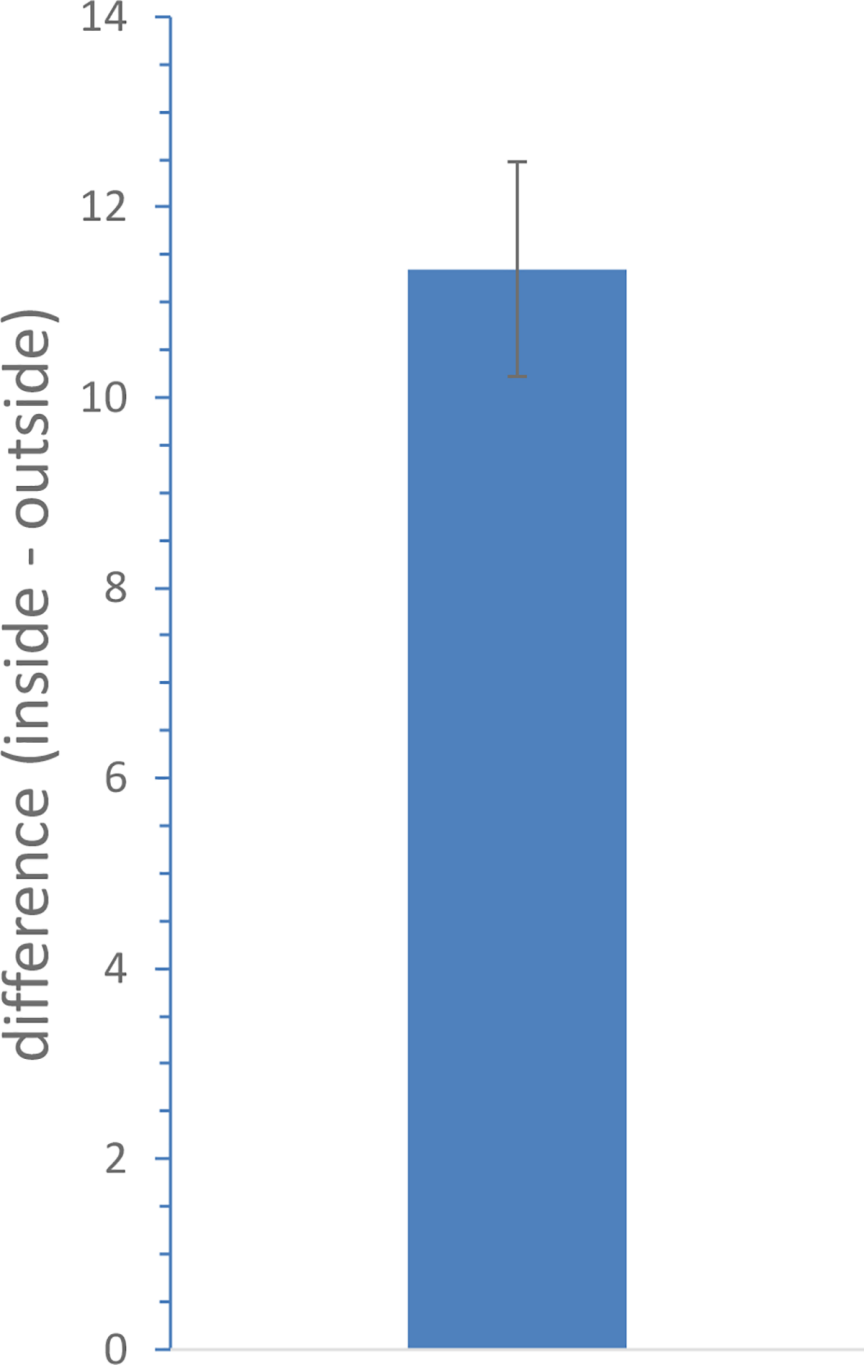
Mean of the difference in predicted species richness between inside and outside each of the Egyptian PAs. This mean is highly significantly different from zero (t323 = 10.1, p<<0.001), and positive, implying that species richness is higher inside than outside the PAs.

The predicted species richness is significantly higher inside PAs than outside in all combinations of assumptions (overall mean excess is 10.2 ± 0.6; a one-sample t-test shows this is significantly greater than zero—t_323_ = 10.1, p<<0.001: see Fig 4). The overall mean species richness outside the PAs is 39.1, and hence on average there is a 26% increase inside the PA.

For unlimited dispersal, the mean predicted species-richness excess inside relative to outside PAs declined slightly with time (S6 Fig). With no dispersal, this predicted excess inside relative to outside declined slightly under both scenarios (S7 Fig).

With unlimited dispersal, under the A2 scenario the maximum species gain by 2020 (S8 Fig) was predicted to be at the Mediterranean coast, along both sides of the Suez Gulf, along the Red Sea coast further to the south, and some scattered patches in Sinai. By 2050, the highest predicted gains appeared along the Mediterranean coast and the Qattara Depression, scattered places on both sides of the Suez Gulf and the Red Sea coast. By 2080, the highest species gains were predicted to be along the Mediterranean coast, the Qattara Depression to Wadi El-Natrun, small areas around greater Cairo, and scattered areas along the Red Sea coast. There were no big differences in predicted gains under the B2 scenario (S8 Fig), although the magnitudes were lower.

For unlimited dispersal, under the A2 scenario the maximum species losses by 2020 (S9 Fig) were predicted to be on both sides of the Suez Gulf, along the Red Sea coast, and some small scattered areas in Sinai, east of Cairo and around Ismailia. By 2050, they were predicted to be along the Red Sea coast and areas around greater Cairo, and by 2080, along the Mediterranean coast, north and south Sinai, some scattered areas on both sides of the Suez Gulf, and along the Red Sea coast. Under the B2 scenario, predicted losses were higher but similar in pattern (S9 Fig).

For the no-dispersal assumption, under both scenarios the pattern of predicted losses did not differ much from those for unlimited dispersal. Differences were only apparent in the magnitudes, which were lower under the no-dispersal assumption (S10 Fig).

**Fig 5:**
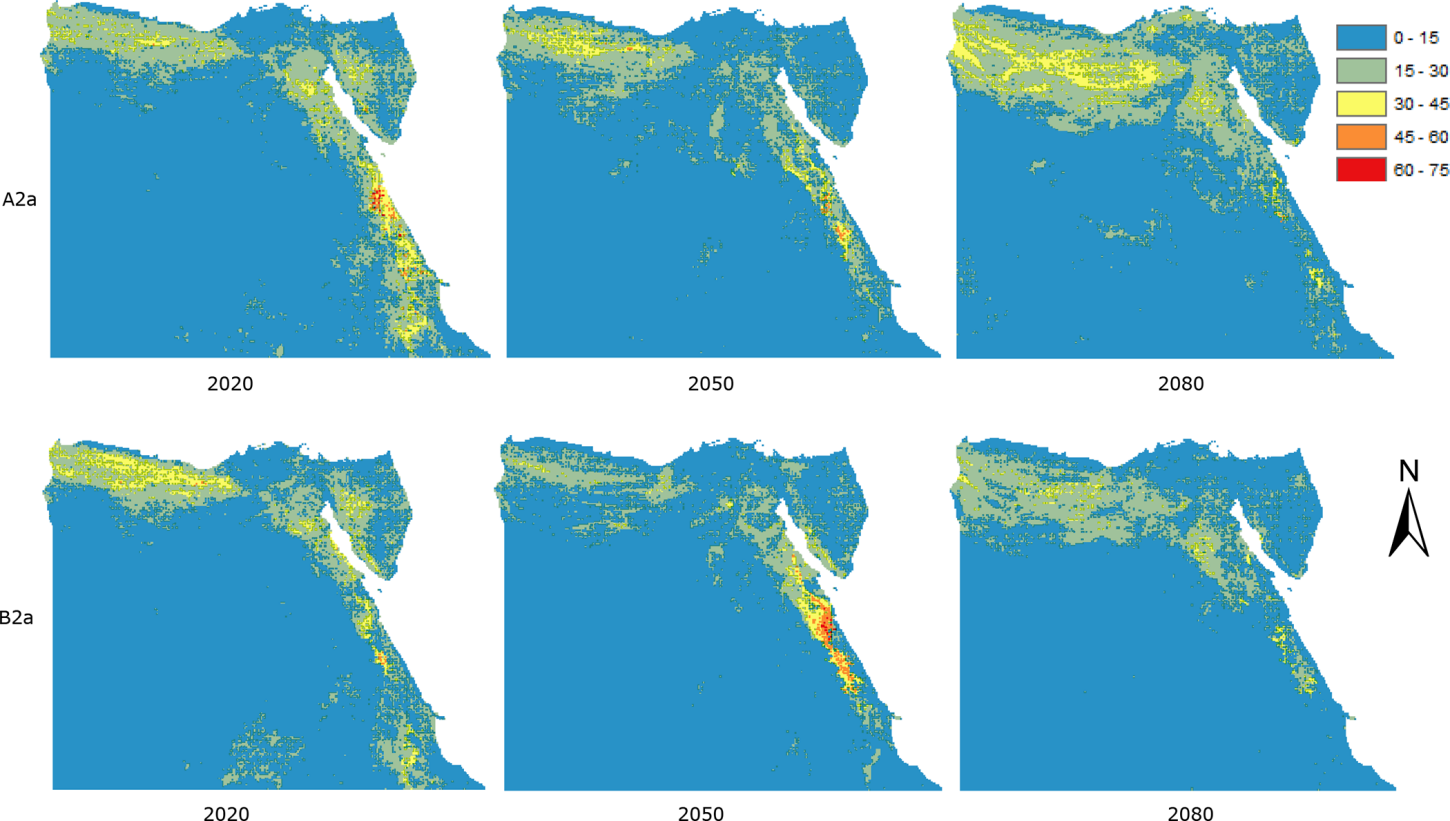
Predicted future species turnover (change in species composition), assuming unlimited dispersal. The colours indicate ranges, from low (blue) to high (red) change in species composition).

For unlimited dispersal, under the A2 scenario by 2020 the highest turnover (Fig 5) was predicted to occur along the Red Sea coast, in Sinai, along the Mediterranean coast, and along both sides of the Suez Gulf. By 2050 the pattern is similar but the magnitude is lower. By 2080, the highest predicted species turnover was along the Mediterranean coast, greater Cairo southwards, scattered areas both sides of the Suez Gulf, and some scattered areas along the Red Sea coast. Under the B2 scenario, the pattern of turnover was very similar (Fig 5), with some differences in magnitude. Under the no-dispersal assumption, turnover for both scenarios and all times were also similar to the pattern of losses under the no-dispersal assumption (see S10 Fig).

## Discussion

The predicted patterns of species richness of Egyptian medicinal plants under climate change and a variety of assumptions showed obvious shifts towards the north and north-east of Egypt, along the Mediterranean coast, Sinai Peninsula, Red Sea coast, and both sides of the Suez Gulf. The most important predictors of these shifts were the temperature-related variables. There are many studies elsewhere demonstrating that species tend to shift poleward in latitude and upwards in elevation [e.g. 3, 8], and the most important drivers for these changes are temperature and rainfall [6]. For example, meta-analysis shows that terrestrial species distributions have shifted about 11 m per decade to higher altitudes, and about 17 km per decade towards higher latitudes, depending on the ability of species to track suitable climate change [56].

Changes in land cover are thought to be one of the critical drivers of biodiversity change [57, 58], but the habitat and NDVI predictors that should reflect it here did not add useful information for the model performance (see S12 Fig, which describes the contribution of the environmental variables). Some studies on arid environment with different taxa have had similar results [36, 59, 60], while others have found that such predictors increased the accuracy of predicted distributions [61, 62]. There are limitations to the use such satellite-derived predictors for species distribution modelling (see [62–64]), but critically we have no models of future scenarios for the habitat and NDVI predictors for Egypt, and thus were unable to include changes in land cover in our modelling. Given Egypt’s rapidly increasing human population, this is the next step to improving predictions of future climate-change effects on biodiversity.

We used only MaxEnt as an SDM method here. Different SDM methods can lead to the same performance in predicting distributions (in terms of the indicators AUC and TSS [50]), including for climate change scenarios [27], but their predictions can vary [65–67]. Ensemble methods that average across the results of different methodologies have been advocated to reduce model uncertainty [68–71], but sometimes this is not an automatic outcome and care is needed [72, 73] to avoid inappropriate conclusions [74].

The predicted species richness gradually increased over time under different scenarios compared with the present day when unlimited dispersal was assumed, because species can then track their habitat under climate change and find suitable environments. Temperatures at the Mediterranean coast, Sinai, the Red Sea coast, and both sides of the Suez Gulf are lower, and rainfall is higher, than in the Western and Eastern deserts and the southern parts of Egypt; these factors create suitable habitat. However, when no-dispersal was assumed, habitat suitability generally declined for future times and climate scenarios compared with current conditions, because species were then not able to track the changing environment. The pattern of species richness appeared to increase more under the A2 scenario than the B2 scenario, as [75] also found.

Notably, Egypt has a hyper-arid environment and enormous desert spaces in the western, eastern and southern regions. Visually there is agreement between plots of the actual records and predictions of species richness under current conditions. Despite now being accessible to modern vehicles, there are very few records found in either desert or southern areas of Egypt, and hence greater species richness northward, and in Sinai and the Red Sea coast. The main reasons behind this low species richness in much of Egypt must lie in the extreme climatic and environmental conditions which are not suitable for most plants (although lack of sampling clearly contributes, especially in the militarily inaccessible Gebel Elba region that is known to be rich in plant and animal species).

There is no previous map, atlas or study about Egyptian plant distributions under climate change with which to compare, but our results are similar to other studies recently conducted for Egyptian mammals, butterflies and reptiles [28, 36, 76]. These all used a similar approach with species distribution modelling, finding that the pattern of species richness increased towards the north and east, with the highest species richness along the Red Sea coast down to Gebel Elba, Sinai, and from the Delta stretching along the Mediterranean coast. Butterflies are good indicators of general biodiversity [76], since they depend on plants throughout their life cycle. The pattern of our results is certainly in agreement with their findings. For mammals [77], there is an even greater concentration of species in these places. All these studies taken together indicate that there will be increases in habitat suitability in the northern and eastern coastal areas of Egypt under climate change, while declines occur in the deserts and southern areas of Egypt.

There were differences between the A2 and B2 scenarios in species gains, losses, and turnover for different future times. Mean species gains under the A2 scenario assuming unlimited dispersal increased more than under the B2 scenario, but the gains declined from 2020 to 2050 and then increased again between 2050 and 2080 for both scenarios. The A2 scenario also had more species losses than the B2 scenario (S8 Fig, S3 Table) under both unlimited and no-dispersal assumptions. Thuiller [5] also suggested that the A2 scenario in some cases might result in higher species richness than the B2 scenario. Similarly, there was greater species turnover in the A2 than the B2 scenario; under both scenarios there was a decline in species turnover between 2020 and 2050, and then an increase again from 2050 to 2080. In general the species losses, gains, and turnover were predicted to happen along the Mediterranean coast, both sides of the Suez Gulf, the Red Sea coast, Qattara Depression, Siwa Oasis, some patches around greater Cairo and Sinai.

Why did the A2 scenario result in higher species richness, even though this is the scenario with maximum climate change? According to the special report on emission scenarios [47], under the A2 scenario there is more climate change related to CO_2_, N_2_O, methane, and other gases than under the B2 scenario (see S1 and S2 Tables). All these gases increase gradually in both scenarios until 2100, and then some decline afterwards, but mostly they remain steady. Under the A2 scenario more of these gases are released than under the B2 scenario. The main SDM predictors in our results are temperature and to a lesser extent precipitation. All environmental variables related to temperature (S4 Table) increased gradually with time under both scenarios, but by 2050 A2 predicted wetter conditions than B2. This may be the reason for the predictions for the A2 scenario.

Other studies from different continents are relevant here. Under the effects of climate change, 2–40% of woody plants in Yunnan Province (China) were predicted to become seriously endangered or disappear by 2080 under the two assumptions of dispersal [78]. Two-thirds of plant species were predicted to decline, and between 5 and 25% to lose their range completely by 2080 in southwestern Australia [79]. Thuiller et al.[44] predicted that more than 50% of 1350 European plants would be under threat by 2080. Golicher et al. [80] suggested that predicted increases in temperature of 3°C and decreases in precipitation by about 20% would cause a decline of about 15% in the species richness of the tropical forests of Mesoamerica. Thus most studies have predicted much more change, and more serious losses, than we do for Egypt.

Protected areas are important strategic elements of conservation planning. Compared with other countries, the proportion of Egypt’s land surface represented by PAs (about 15%) is higher, but as elsewhere, there was no consideration of the potential effects of climate change when these PAs were selected [81]. With the first being established in 1983, Egyptian PAs are all relatively new, but they were chosen under scientific supervision and using expert knowledge about Egyptian biodiversity [28]. The likely future species richness of medicinal plants inside PAs was significantly higher than outside for all combinations of assumptions. These results suggest that Egyptian PAs are placed appropriately for medicinal plants, because they provide suitable environments for them to flourish under climate change, and also effectively represent the diversity of the country. We would argue that Egypt's medicinal plants (which include many shrubs and woody perennials) are representative of plants in general and the lack of suitably validated data for most other plants precluded their use here.

However, there are many locations outside PAs that have high species richness, and hence this needs more effort and management to provide suitable habitat to avoid losing particular species in the future. This is particularly true of areas along the Red Sea coast, some locations around the Suez Gulf, and areas around the Qattara Depression. This result concurs with other studies conducted on other Egyptian taxonomic groups [28, 76]. Leach et al. [33] found that the present-day species richness of butterflies and mammals was higher inside than outside PAs, while in the future this difference will not be stable, as was found for other taxonomic groups in other regions [81, 82]. It is important to make this comparison to facilitate assessing the relative contribution of PAs to the protection of regional biodiversity [81]. Therefore, we conclude that SDM approaches can be used as guidelines for managing PAs or other environments [22, 83].

Species richness is just one of the criteria to assess PAs in conservation planning [28]. Alternative measures are possible; Kershaw et al.[84] combined biotic diversity, species uniqueness, and the degree of endangerment, while [85] focussed on specific threats such as land acquisition and invasive species. There are many other factors that impact on species richness distribution and species losses inside and outside PAs, for instance, increasing human land use [86, 87] or plant productivity [88]. Botkin et al. [9] suggested that the interaction between climate change and resource use by humans could have undesirable effects on biodiversity, greater than each of them alone. Therefore it is important for the Egyptian government to take quick steps to allow for all the possibilities of habitat loss in the future. Genetic erosion may happen in some species, and we must pre-empt the possibility of future extinctions by collecting and preserving seeds.

Overall it is clear that even a modest quantity of data can produce informative and useful guidance for the likely effects of climate change. The first step is the collation, checking and updating of records into a usable database; nearly all countries can do this. Once done, studies such as ours can suggest reasonable and evidence-based precautionary measures that can be taken to mitigate climate change.

## Supporting information

S1 Fig An example to show predicted gain and loss areas in the future distribution of one species. (PDF)

S2 Fig Potential effect of different climate change scenarios (A2a and B2a) on changes in future species richness (using binary species distributions and assuming unlimited dispersal); maps created as differences between future and current species richness maps (presented in Fig 2). Red means increasing future species richness, and blue means declining future species richness.
(PDF)

S3 Fig Potential effect of different climate change scenarios (A2a and B2a) on changes in future species richness (using binary species distribution and assuming no-dispersal); maps created as differences between future and current species richness maps (presented in Fig 3). Red means decline future species richness, and blue means increasing future species richness.
(PDF)

S4 Fig Average predicted mean species richness assuming unlimited dispersal for binary distributions, A): Current and future times with the A2a scenario; B): current and future times with the B2a scenario.
(PDF)

S5 Fig Predicted mean species richness assuming no-limited dispersal for binary distributions for the two scenarios A2a (left) and B2a (right). (PDF)

S6 Fig Species richness excess inside relative to outside PAs through time, using binary distributions (both assuming unlimited dispersal). A): A2a scenario; B): B2a scenario.
(PDF)

S7 Fig Species richness excess inside relative to outside PAs using binary distribution (assuming no dispersal). (PDF)

S8 Fig Spatial pattern of the number of species gained under climate change in the future (assuming unlimited dispersal). Colours indicate few (blue) to many (red) species gained.
(PDF)

S9 Fig Spatial pattern of the number of species lost under climate change in the future (assuming unlimited dispersal). Colours indicate few (blue) to many (red) species lost.
(PDF)

S10 Fig Spatial pattern of the number of species lost under climate change in the future (assuming no dispersal). Colours indicate few (blue) to many (red) species lost.
(PDF)

S11 Fig Egypt’s political border and all cities and geographical regions mentioned in this study (El-Gabbas et al., 2016). (PDF)

S12 Fig Contribution to the final species distribution models made by each environmental predictor, illustrated by the mean permutation importance (Kaky and Gilbert, 2016). (PDF)

S1 Table Overview of greenhouse gas emissions between 1990–2100, modified from the IPCC special report on emission scenarios. Units are given in the table.
(PDF)

S2 Table Standardized anthropogenic emissions (CO_2_, N_2_O, CH_4_, and NO_x_) for the ALM region (Africa and Latin America), the region containing our study area; modified from (Nakicenovic *et al*., 2000) Units given in the table. (PDF)

S3 Table Mean number of species gained, lost, and turnover under both dispersal assumptions (unlimited and no dispersal) for both scenarios (A2 and B2) at various future times (2020, 2050, and 2080). (PDF)

S4 Table Mean value for all environmental variables related to temperature and precipitation. (PDF)

S5 Table List of the plant species and their status, with the number of the records within Egypt. (PDF)

S6 Table The main sources of the location database of medicinal plants, put together by the BioMAP project 2004–8. Many of the records upon which the MSc and PhD theses are based are in the main Cairo Herbarium, but they declined to give access to their database without payment. The BioMAP team of taxonomists included the organisers of the OpWall expeditions and the Medicinal Plants project, hence their access to these records.
(PDF)
