# Supplementary figures and images for: Predicting the distributions of Egypt's medicinal plants and their potential shifts under future climate change
Source: PLoS One. 2017 Nov 14;12(11):e0187714. doi: 10.1371/journal.pone.0187714 (PMC5685616; doi:10.1371/journal.pone.0187714)

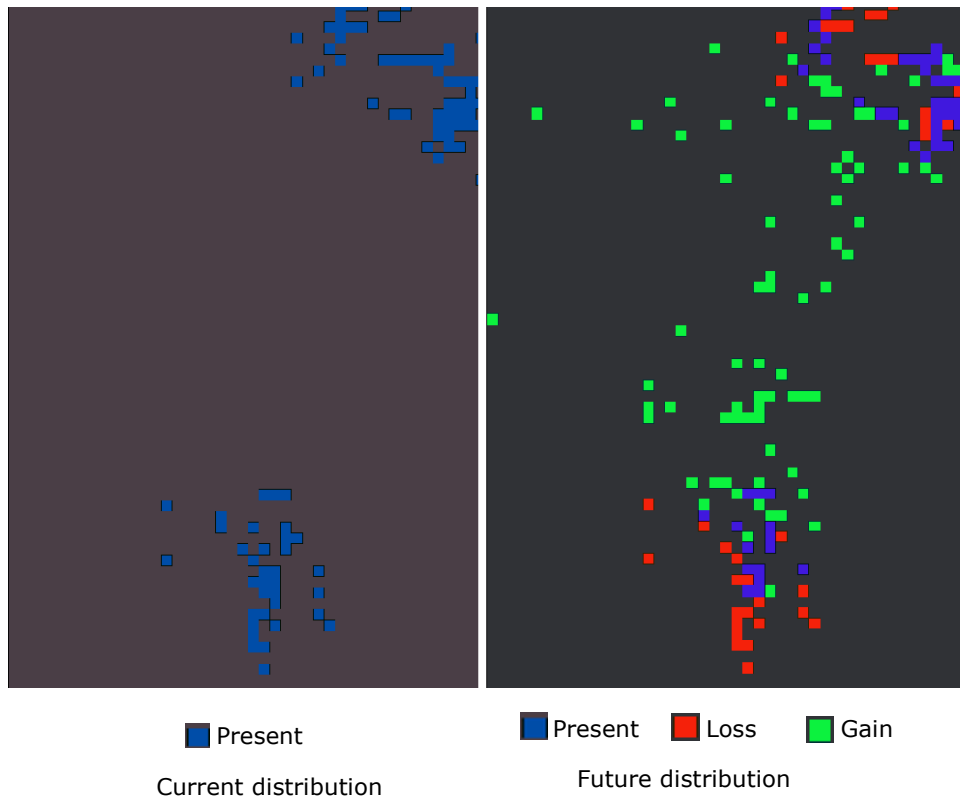

**S1 Fig.** An example to show predicted gain and loss areas in the future distribution of one species.

Supplement: S1 Fig — (PDF) [file pone.0187714.s001.pdf]
