# Supplementary material for: Predicting the distributions of Egypt's medicinal plants and their potential shifts under future climate change
Source: PLoS One. 2017 Nov 14;12(11):e0187714. doi: 10.1371/journal.pone.0187714 (PMC5685616; doi:10.1371/journal.pone.0187714)
Supplement: S2 Fig — Red means increasing future species richness, and blue means declining future species richness. (PDF) [file pone.0187714.s002.pdf]

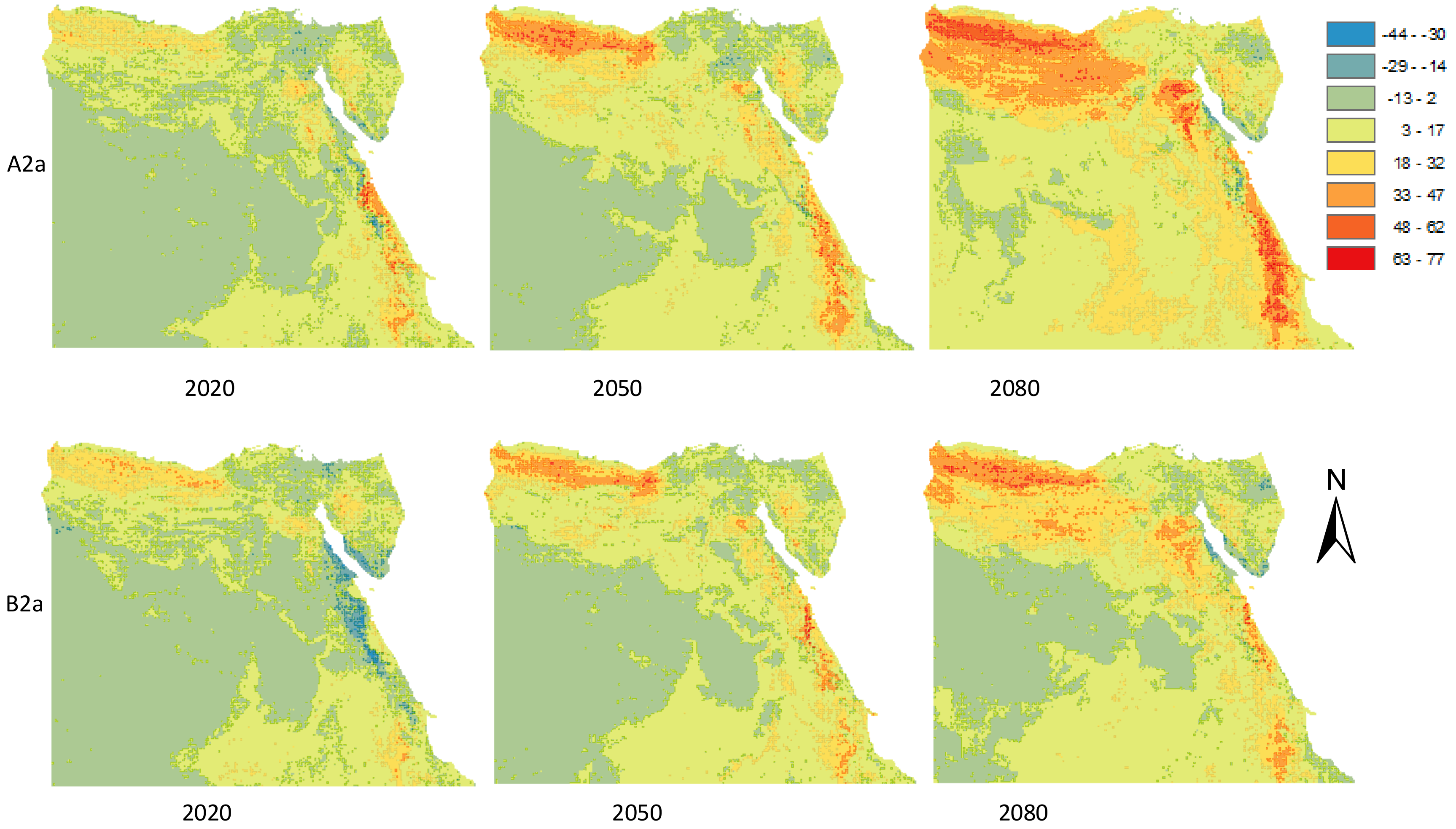

S2 Fig. Potential effect of different climate change scenarios (A2a and B2a) on changes in future species richness (using binary species distributions and assuming unlimited dispersal); maps created as differences between future and current species richness maps (presented in Fig 2). Red means increasing future species richness, and blue means declining future species richness.
