# Supplementary material for: Predicting the distributions of Egypt's medicinal plants and their potential shifts under future climate change
Source: PLoS One. 2017 Nov 14;12(11):e0187714. doi: 10.1371/journal.pone.0187714 (PMC5685616; doi:10.1371/journal.pone.0187714)
Supplement: S4 Fig — Average predicted mean species richness assuming unlimited dispersal for binary distributions, A): Current and future times with the A2a scenario; B): current and future times with the B2a scenario. (PDF) [file pone.0187714.s004.pdf]

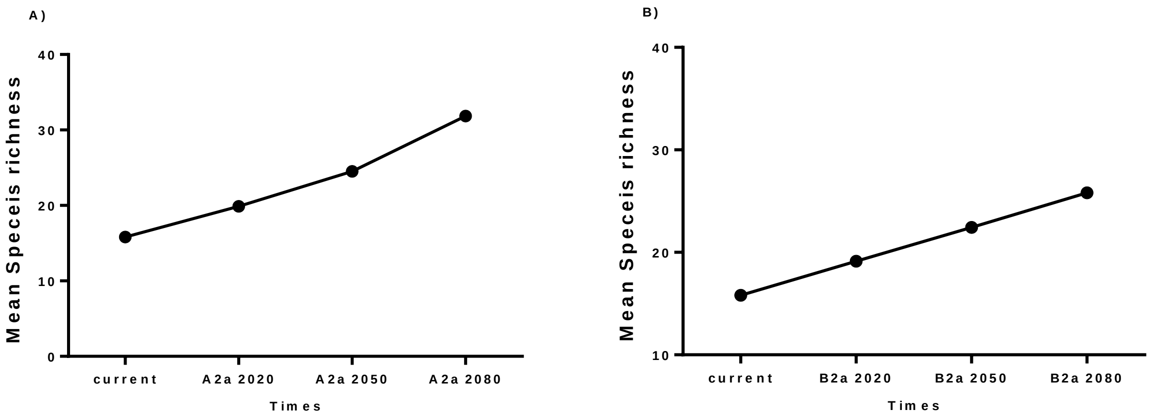

S4 Fig. Average predicted mean species richness assuming unlimited dispersal for binary distributions, A): Current and future times with the A2a scenario; B): current and future times with the B2a scenario.
