# Supplementary material for: Predicting the distributions of Egypt's medicinal plants and their potential shifts under future climate change
Source: PLoS One. 2017 Nov 14;12(11):e0187714. doi: 10.1371/journal.pone.0187714 (PMC5685616; doi:10.1371/journal.pone.0187714)
Supplement: S5 Fig — (PDF) [file pone.0187714.s005.pdf]

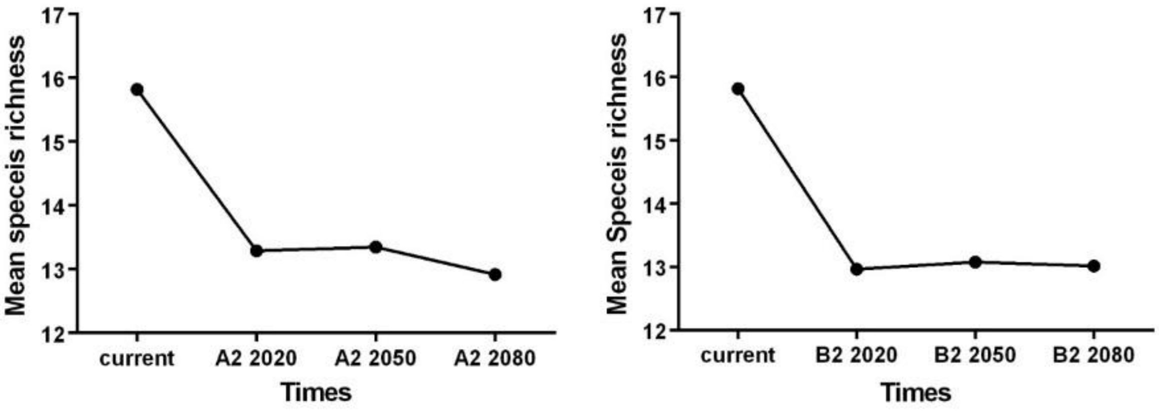

S5 Fig. Predicted mean species richness assuming no-limited dispersal for binary distributions for the two scenarios A2a (left) and B2a (right).
