# Supplementary material for: Predicting the distributions of Egypt's medicinal plants and their potential shifts under future climate change
Source: PLoS One. 2017 Nov 14;12(11):e0187714. doi: 10.1371/journal.pone.0187714 (PMC5685616; doi:10.1371/journal.pone.0187714)
Supplement: S6 Fig — A): A2a scenario; B): B2a scenario. (PDF) [file pone.0187714.s006.pdf]

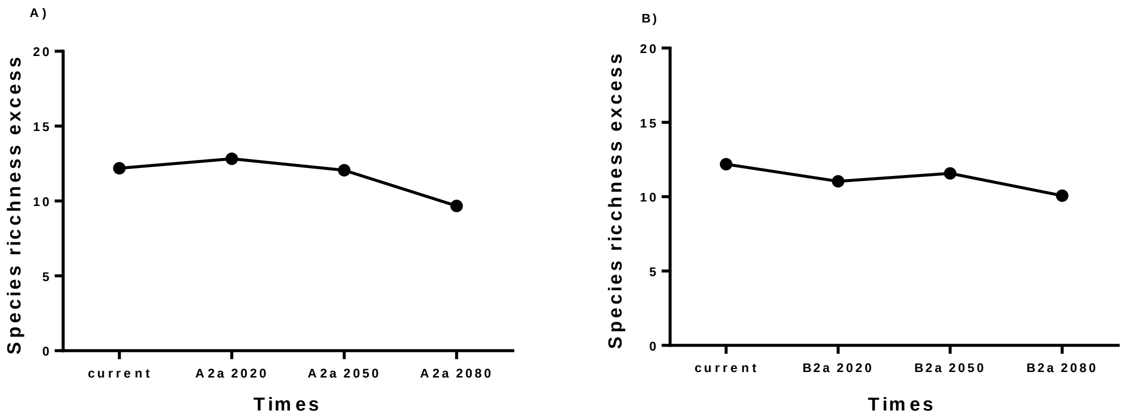

S6 Fig. Species richness excess inside relative to outside PAs through time, using binary distributions (both assuming unlimited dispersal). A): A2a scenario; B): B2a scenario.
