# Supplementary material for: Predicting the distributions of Egypt's medicinal plants and their potential shifts under future climate change
Source: PLoS One. 2017 Nov 14;12(11):e0187714. doi: 10.1371/journal.pone.0187714 (PMC5685616; doi:10.1371/journal.pone.0187714)
Supplement: S8 Fig — Colours indicate few (blue) to many (red) species gained. (PDF) [file pone.0187714.s008.pdf]

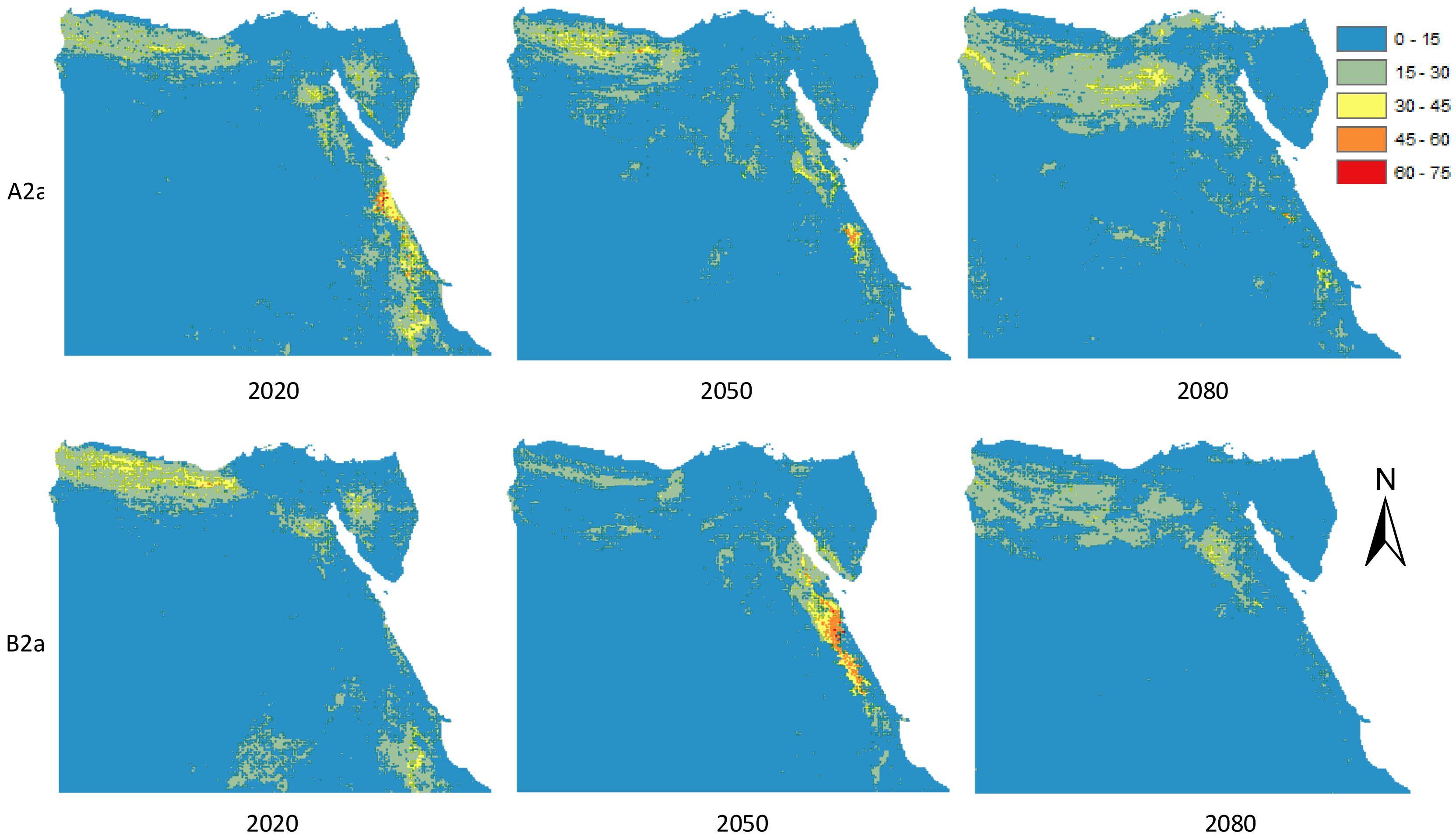

S8 Fig. Spatial pattern of the number of species gained under climate change in the future (assuming unlimited dispersal). Colours indicate few (blue) to many (red) species gained.
