# Supplementary material for: Predicting the distributions of Egypt's medicinal plants and their potential shifts under future climate change
Source: PLoS One. 2017 Nov 14;12(11):e0187714. doi: 10.1371/journal.pone.0187714 (PMC5685616; doi:10.1371/journal.pone.0187714)
Supplement: S10 Fig — Colours indicate few (blue) to many (red) species lost. (PDF) [file pone.0187714.s010.pdf]

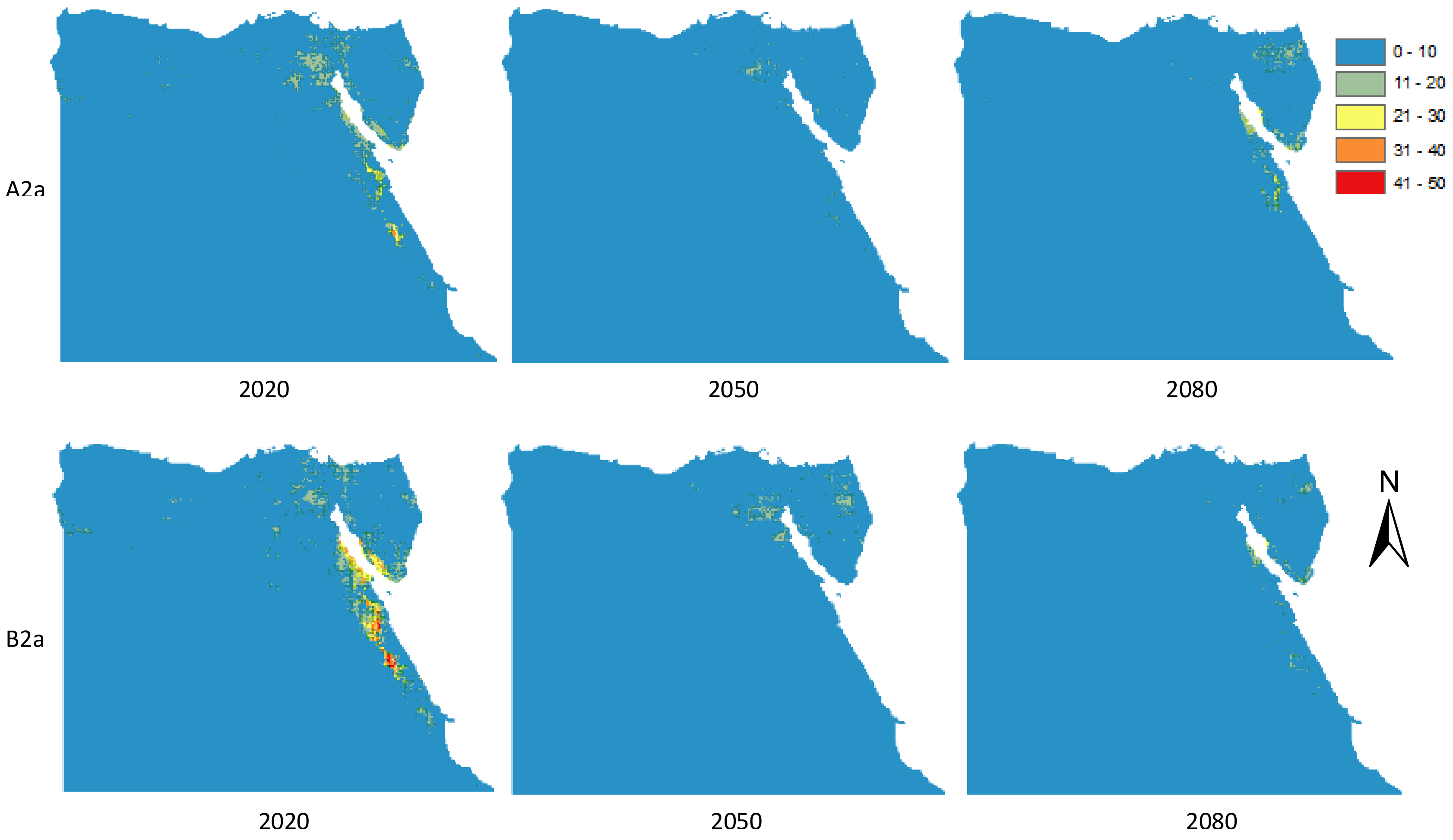

S10 Fig. Spatial pattern of the number of species lost under climate change in the future (assuming no dispersal). Colours indicate few (blue) to many (red) species lost.
