# Supplementary material for: Predicting the distributions of Egypt's medicinal plants and their potential shifts under future climate change
Source: PLoS One. 2017 Nov 14;12(11):e0187714. doi: 10.1371/journal.pone.0187714 (PMC5685616; doi:10.1371/journal.pone.0187714)
Supplement: S11 Fig — (PDF) [file pone.0187714.s011.pdf]

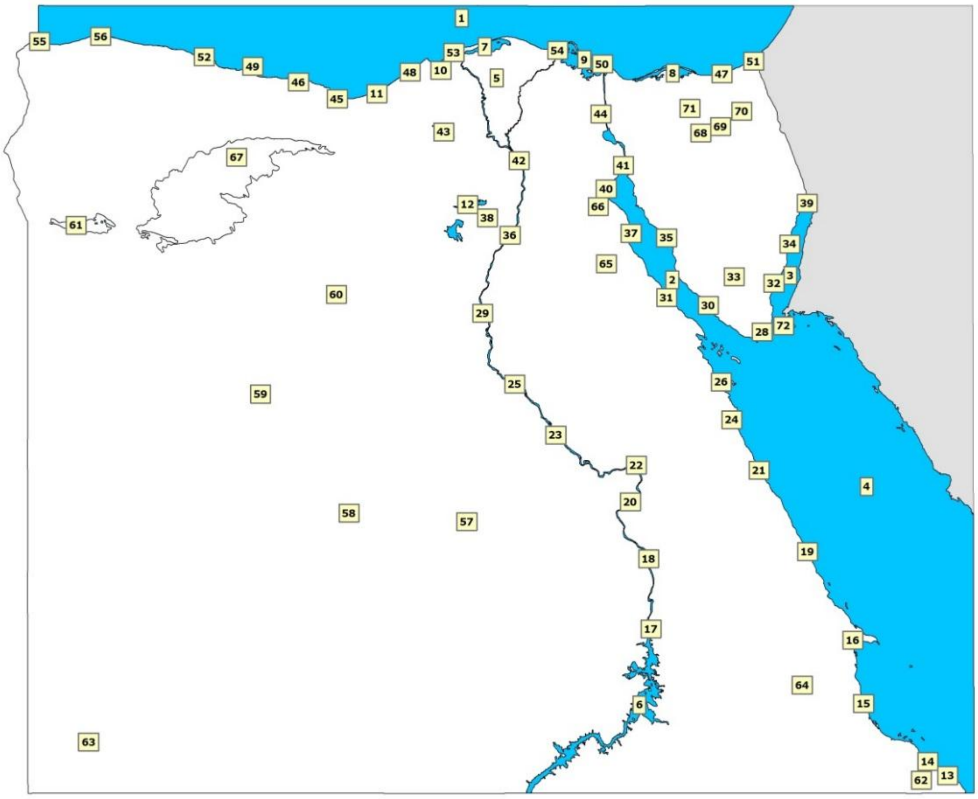

**1**	The Mediterranean Sea	**25**	Assiut	**49**	Ras El-Hekma
**2**	The Suez Gulf	**26**	Hurghada	**50**	Port-Said
**3**	The Aqaba Gulf	**27**	Ras Mohamed	**51**	Rafah
**4**	The Red Sea	**28**	Sharm El-Sheikh	**52**	Mersa Matruh
**5**	The Nile Delta	**29**	El-Minia	**53**	Rosetta
**6**	Lake Nasser	**30**	El-Tur	**54**	Damietta
**7**	Lake Brullus	**31**	Ras Gharib	**55**	Sallum
**8**	Lake Bardawil	**32**	Dahab	**56**	Sidi Barrani
**9**	Lake Manzala	**33**	Saint-Katherine	**57**	Kharga oasis
**10**	Lake Idku	**34**	Nuweiba	**58**	Dakhla oasis
**11**	Lake Mariut	**35**	Abu Zneima	**59**	Farafra oasis
**12**	Lake Qarun	**36**	Beni Suef	**60**	Bahariya oasis
**13**	Halayeb	**37**	Ras Zaafarana	**61**	Siwa oasis
**14**	Abu Ramad	**38**	Fayoum	**62**	Gebel Elba area
**15**	Al-Shalatein	**39**	Taba	**63**	El-Gilf El-Kebir
**16**	Berenice	**40**	Ain Sukhna	**64**	Gebel Abraq area
**17**	Aswan	**41**	Suez	**65**	Gebel El-Gallala El-Qibliya
**18**	Edfu	**42**	The greater Cairo	**66**	Gebel El-Gallala El-Bahariya
**19**	Mersa Alam	**43**	Wadi El-Natrun	**67**	Qattara Depression
**20**	Luxor	**44**	Ismailia	**68**	Gebel Yillaq
**21**	El-Quseir	**45**	El-Alamein	**69**	El-Hassana
**22**	Qena	**46**	El-Dabaa	**70**	Gebel El-Hallal
**23**	Sohag	**47**	El-Arish	**71**	Gebel El-Maghara
**24**	Safaga	**48**	Alexandria	**72**	Tiran & Sanafir islands

S11 Fig. Egypt's political border and all cities and geographical regions mentioned in this study (El-Gabbas et al., 2016).
