# Supplementary material for: Predicting the distributions of Egypt's medicinal plants and their potential shifts under future climate change
Source: PLoS One. 2017 Nov 14;12(11):e0187714. doi: 10.1371/journal.pone.0187714 (PMC5685616; doi:10.1371/journal.pone.0187714)
Supplement: S12 Fig — (PDF) [file pone.0187714.s012.pdf]

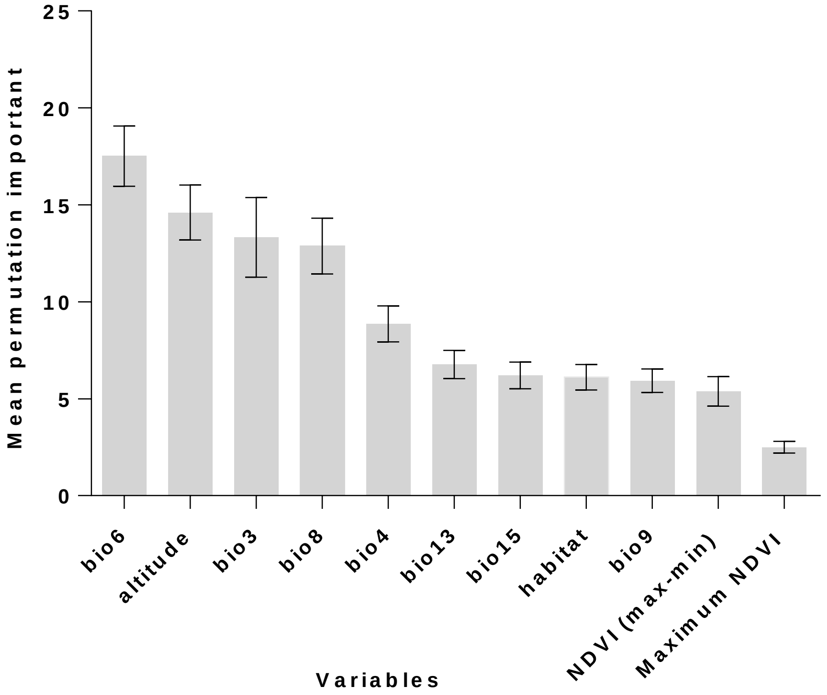

S12 Fig. Contribution to the final species distribution models made by each environmental predictor, illustrated by the mean permutation importance (Kaky and Gilbert, 2016).
