# Supplementary material for: Predicting the distributions of Egypt's medicinal plants and their potential shifts under future climate change
Source: PLoS One. 2017 Nov 14;12(11):e0187714. doi: 10.1371/journal.pone.0187714 (PMC5685616; doi:10.1371/journal.pone.0187714)
Supplement: S1 Table — Units are given in the table. (PDF) [file pone.0187714.s013.pdf]

S1 Table. Overview of greenhouse gas emissions between 1990 – 2100, modified from the IPCC special report on emission scenarios (Nakicenovic *et al.*, 2000). Units are given in the table.

Gases	Current	Scenario group					
		A2			B2		
	1990	2020	2050	2100	2020	2050	2100
Carbon dioxide, fossil fuels (GtC/yr)	6	11	16.5	28.9	9	11.2	13.8
Carbon dioxide, land use (GtC/yr)	1.1	1.2	0.9	0.2	0	-0.2	-0.5
Cumulative carbon dioxide, fossil fuels (GtC) 1990-2100		1773			1160		
Cumulative carbon dioxide, land use (GtC) 1990-2100		89			4		
Cumulative carbon dioxide, total (GtC) 1990-2100		1862			1164		
Methane ($MtCH_4$/yr)	310	424	598	889	384	505	597
Nitrous oxide (MtN/yr)	6.7	9.6	12	16.5	6.1	6.3	6.9
NO_x (MtN/yr)	30.9	50	71	109	43	55	61
