# Supplementary material for: Predicting the distributions of Egypt's medicinal plants and their potential shifts under future climate change
Source: PLoS One. 2017 Nov 14;12(11):e0187714. doi: 10.1371/journal.pone.0187714 (PMC5685616; doi:10.1371/journal.pone.0187714)
Supplement: S2 Table — (PDF) [file pone.0187714.s014.pdf]

S2 Table. Standardized anthropogenic emissions (CO_2, N_2O, CH_4, and NO_x) for the ALM region (Africa and Latin America), the region containing our study area; modified from (Nakicenovic *et al.*, 2000) Units given in the table.

Gases	Current	Scenario group					
		A2			B2		
	1990	2020	2050	2100	2020	2050	2100
Carbon dioxide, fossil fuels (GtC/yr)	1.30	1.22	1.52	2.41	0.81	1.24	1.18
Carbon dioxide, land use (GtC/yr)	0.00	0.00	0.00	0.00	-0.18	-0.04	-0.04
Total CO_2	1.30	1.22	1.52	2.41	0.63	1.20	1.14
Methane total ($MtCH_4$/yr)	47.10	45.80	78.00	143.20	39.80	52.90	47.00
Nitrous oxide total (MtN/yr)	0.60	0.70	0.80	1.00	0.60	0.60	0.70
NO_x (MtN/yr)	4.70	4.00	5.30	7.60	3.20	5.20	3.70
