# Supplementary material for: Predicting the distributions of Egypt's medicinal plants and their potential shifts under future climate change
Source: PLoS One. 2017 Nov 14;12(11):e0187714. doi: 10.1371/journal.pone.0187714 (PMC5685616; doi:10.1371/journal.pone.0187714)
Supplement: S3 Table — (PDF) [file pone.0187714.s015.pdf]

S3 Table. Mean number of species gained, lost, and turnover under both dispersal assumptions (unlimited and no dispersal) for both scenarios (A2 and B2) at various future times (2020, 2050, and 2080).

**Measurements**	**Unlimited dispersal**						**No dispersal**					
	**A2 Scenario**			**B2 Scenario**			**A2 Scenario**			**B2 Scenario**		
	**2020**	2050	**2080**	**2020**	**2050**	**2080**	**2020**	**2050**	**2080**	**2020**	**2050**	**2080**
Mean species gained	7.41	6.44	9.21	6.19	5.37	5.58	There are no species gained under this assumption					
Mean species lost	1.84	1.83	2.85	1.8	2.08	2.22	1.47	0.69	1.07	1.8	1.06	0.77
Mean species turnover	9.24	8.27	12.05	7.99	7.45	7.8	1.47	0.69	1.07	1.8	1.06	0.77
