# Supplementary material for: Predicting the distributions of Egypt's medicinal plants and their potential shifts under future climate change
Source: PLoS One. 2017 Nov 14;12(11):e0187714. doi: 10.1371/journal.pone.0187714 (PMC5685616; doi:10.1371/journal.pone.0187714)
Supplement: S4 Table — (PDF) [file pone.0187714.s016.pdf]

S4 Table. Mean value for all environmental variables related to temperature and precipitation.

Environmental Variables	Current	Scenario groups					
		A2			B2		
		2020	2050	2080	2020	2050	2080
Isothermality (Bio3)	47.93	46.44	46.06	44.63	47.09	46.40	45.70
Temperature seasonality (Bio4)	5843.36	5959.45	6051.81	6378.62	5868.28	6063.21	6207.14
Minimum temperature of the coldest month (Bio6)	57.58	67.63	82.48	98.58	71.21	80.12	87.41
Mean temperature of the wettest quarter (Bio8)	169.95	173.86	189.36	202.97	182.35	184.74	194.62
Mean temperature of the driest quarter (Bio9)	200.94	199.16	218.48	224.28	199.22	213.3	218.4
precipitation of the wettest month (Bio13)	3.83	3.37	3.56	3.21	3.42	3.25	3.20
precipitation seasonality (Bio15)	45.19	36.77	42.44	40.41	40.21	36.18	40.53
