# Supplementary material for: Predicting the distributions of Egypt's medicinal plants and their potential shifts under future climate change
Source: PLoS One. 2017 Nov 14;12(11):e0187714. doi: 10.1371/journal.pone.0187714 (PMC5685616; doi:10.1371/journal.pone.0187714)
Supplement: S5 Table — (PDF) [file pone.0187714.s017.pdf]

S5 Table. List of the plant species and their status, with the number of the records within Egypt.

Speceis	Native	World Status	Egypt Status	Number of records
Acacia pachyceras	native	restricted	localized	11
Acacia tortilis	native	narrow	widespread	242
Achillea fragrantissima	native	restricted	widespread	239
Achillea santolina	native	narrow	widespread	13
Adonis dentata	native	narrow	narrow	33
Aerva javanica	native	widespread	widespread	175
Agathophora alopecuroides	native	narrow	narrow	26
Alhagi graecorum	native	narrow	widespread	100
Anabasis articulata	native	narrow	widespread	126
Anagallis arvensis	native	widespread	widespread	119
Anastatica hierochuntica	native	narrow	widespread	31
Andrachne aspera	native	narrow	widespread	31
Artemisia judaica	native	narrow	widespread	684
Artemisia monosperma	native	restricted	narrow	115
Asclepias sinaica	native	near-endemic	narrow	31
Asparagus stipularis	native	narrow	widespread	20
Atriplex halimus	native	narrow	narrow	125
Avena barbata	native	narrow	widespread	22
Ballota undulata	native	restricted	narrow	124
Bassia muricata	native	narrow	widespread	83
Calendula arvensis	native	widespread	widespread	66
Calotropis procera	native	widespread	widespread	228
Capparis spinosa	native	narrow	localized	241
Chenopodium album	native	widespread	widespread	166
Chenopodium murale	native	widespread	widespread	319
Chiliadenus montanus	native	near-endemic	narrow	88
Citrullus colocynthis	native	narrow	widespread	168
Cleome amblyocarpa	native	narrow	widespread	71
Colutea istria	native	restricted	localized	24
Cornulaca monacantha	native	widespread	widespread	93
Cymbopogon schoenanthus	native	narrow	narrow	15
Cynodon dactylon	native	widespread	widespread	240
Deverra tortuosa	native	restricted	widespread	141
Deverra triradiata	native	restricted	narrow	64
Diplotaxis acris	native	narrow	narrow	60
Diplotaxis erucoides	native	widespread	widespread	12
Diplotaxis harra	native	restricted	narrow	147
Echinops spinosus	native	narrow	widespread	213
Ephedra alata	native	narrow	widespread	46
Eruca sativa	non-native	widespread	widespread	96
Euphorbia peplis	native	narrow	narrow	13
Euphorbia retusa	native	narrow	narrow	114
Fagonia arabica	native	restricted	widespread	294

Fagonia glutinosa	native	narrow	narrow	125
Fagonia mollis	native	near-endemic	narrow	698
Farsetia aegyptia	native	narrow	widespread	114
Globularia arabica	native	restricted	narrow	51
Gypsophila capillaris	native	narrow	localized	59
Halocnemum strobilaceum	native	widespread	widespread	53
Haloxylon salicornicum	native	narrow	widespread	242
Haloxylon scoparium	native	narrow	narrow	36
Haplophyllum tuberculatum	native	narrow	widespread	73
Heliotropium arbainese	native	narrow	widespread	146
Herniaria hirsuta	native	widespread	widespread	13
Hyoscyamus muticus	native	narrow	widespread	143
Imperata cylindrica	native	widespread	widespread	109
Iphiona mucronata	native	near-endemic	narrow	53
Juncus rigidus	native	narrow	widespread	235
Lavandula pubescens	native	restricted	narrow	15
Lycium shawii	native	restricted	widespread	161
Malva parviflora	native	widespread	widespread	165
Melilotus indicus	native	widespread	widespread	239
Mesembryanthemum crystallinum	native	widespread	widespread	38
Mesembryanthemum forsskaolli	native	restricted	widespread	13
Mesembryanthemum nodiflorum	native	narrow	narrow	79
Moltkiopsis ciliata	native	narrow	widespread	181
Nitraria retusa	native	narrow	widespread	100
Noaea mucronata	native	narrow	narrow	47
Ochradenus baccatus	native	narrow	widespread	137
Orobanche cernua	native	widespread	widespread	42
Pancratium sickenbergeri	native	near-endemic	narrow	42
Panicum turgidum	native	narrow	widespread	164
Paronychia arabica	native	narrow	narrow	169
Paronychia argentea	native	narrow	narrow	17
Peganum harmala	native	widespread	narrow	138
Pergularia tomentosa	native	narrow	widespread	362
Phoenix dactylifera	native	widespread	widespread	64
Phragmites australis	native	narrow	narrow	128
Plantago afra	native	narrow	widespread	43
Plantago ovata	native	narrow	widespread	36
Pluchea dioscoridis	native	narrow	widespread	62
Polycarpaea repens	native	narrow	narrow	172
Polycarpon succulentum	native	restricted	widespread	94
Pulicaria undulata	native	narrow	widespread	271
Reaumuria hirtella	native	restricted	localized	109
Reichardia tingitana	native	widespread	widespread	120
Retama raetam	native	narrow	narrow	261
Salvia aegyptiaca	native	narrow	widespread	69
Salvia lanigera	native	narrow	narrow	44

Senecio glaucus	native	narrow	widespread	159
Seriphidium herba-album	native	narrow	widespread	267
Silene succulenta	native	restricted	localized	25
Silene villosa	native	narrow	widespread	51
Sisymbrium irio	native	widespread	widespread	56
Solanum elaeagnifolium	non-native	widespread	narrow	24
Solanum nigrum	native	endemic	localized	199
Stachys aegyptiaca	native	near-endemic	narrow	168
Stipagrostis scoparia	native	restricted	widespread	49
Tamarix aphylla	native	narrow	widespread	86
Tamarix nilotica	native	narrow	widespread	332
Tephrosia purpurea	native	near-endemic	widespread	78
Teucrium leucocladum	native	near-endemic	narrow	66
Teucrium polium	native	narrow	narrow	133
Thymelaea hirsuta	native	narrow	widespread	61
Tribulus terrestris	native	widespread	widespread	65
Trifolium resupinatum	native	narrow	widespread	126
Trigonella stellata	native	narrow	widespread	62
Urginea maritima	native	narrow	narrow	24
Urtica urens	native	widespread	widespread	34
Vicia sativa	native	widespread	localized	68
Zilla spinosa	native	narrow	widespread	632
Zygophyllum album	native	narrow	widespread	115
Zygophyllum coccineum	native	near-endemic	widespread	488
Zygophyllum dumosum	native	near-endemic	narrow	27
