# Supplementary material for: Predicting the distributions of Egypt's medicinal plants and their potential shifts under future climate change
Source: PLoS One. 2017 Nov 14;12(11):e0187714. doi: 10.1371/journal.pone.0187714 (PMC5685616; doi:10.1371/journal.pone.0187714)
Supplement: S6 Table — Many of the records upon which the MSc and PhD theses are based are in the main Cairo Herbarium, but they declined to give access to their database without payment. The BioMAP team of taxonomists included the organisers of the OpWall expeditions and the Medicinal Plants project, hence their access to these records. (PDF) [file pone.0187714.s018.pdf]

S6 Table. The main sources of the location database of medicinal plants, put together by the BioMAP project 2004-8. Many of the records upon which the MSc and PhD theses are based are in the main Cairo Herbarium, but they declined to give access to their database without payment. The BioMAP team of taxonomists included the organisers of the OpWall expeditions and the Medicinal Plants project, hence their access to these records.

Number of records	Source			
1950	Opwall	2008	Operation Wallacea Sinai expedition report, unpublished	
888	Gibali MAA	1988	Studies on the Flora of Northern Sinai	MSc Thesis, Faculty of Science, Cairo University, 403 pp.
598	El Bakry EAH	1982	Studies on Plant Life in the Cairo-Ismailia Region	MSc Thesis, Cairo University
591	North Sinai Medicinal Plants Project	2004	unpublished report	
521	Marie AH	2000	A model for the use of GIS in vegetation mapping of a phytogeographical region …	PhD Thesis, Faculty of Science, Al-Azhar Univerity, Cairo
494	Täckholm V	1969	Alfred Kaiser's Sinai-Herbarium	Publications of the Cairo University Herbarium, Giza. 181 pp.
437	Fayed AAA et al	2004	Flora of Saint Katherine Protectorate	UNEP Conservation & Sustainable Use of Medicinal Plants in Egypt, unpublished report
422	Abdallah MA et al	1984	Materials from the CAIM Herbarium, 2nd ed. Flora of the Sinai peninsula	Cairo, Egypt
379	Opwall	2007	Operation Wallacea Sinai expedition report, unpublished	
361	Hosni AI	1984	Taxonomic revision of the species of Caryophyllaceae in Egypt	PhD Thesis, Cairo University
339	El-Gazzar A	1962-2006	private database	
286	Abd El_Wahab RH et al	2008	Diversity and distribution of medicinal plants in North Sinai, Egypt	African Journal of Environmental Science and Technology 2(7), 157-171
282	Eastern Desert MP project	2000	Medicinal plants of the Eastern Desert	unpublished report.
278	Abdallah MS et al	1972	Natural flora of Egyptian Nubia before the construction of Aswan High Dam	Ministry of Agriculture, Cairo 83 pp.
276	Al-Gohary IH	2008	Floristic composition of eleven wadis in Gebel Elba, Egypt	International Journal of Agriculture & Biology 10: 151-160
259	Opwall	2005	Vegetation and grazing in the St. Katherine Protectorate, South Sinai, Egypt. Operation Wallacea Sinai expedition report, unpublished	
217	Hosni AI	1978	Revision of the genus Zygophyllum L. , Sections bipartia and mediterranea in Egypt	MSc Thesis, Cairo University

214	El-Gad IAH	1987	Taxonomic Studies Of The Genus Fagonia (Zygophyllaceae) In Egypt	MSc Thesis, Cairo University
214	Safa SAIA	1992	Taxonomic studies in the Chenopodiaceae of Egypt	MSc Thesis, Cairo University
210	El Hadidi AMH	1989	Flora Of Egypt , Systematic Revision of Boraginaceae	PhD Thesis, Cairo University
197	Opwall	2006	Opwall plants report - 2006	OpWall Plant Report
192	Zareh MMM	1984	Taxonomic Studies on the Ganaphaliinae (Asteraceae-Inuleae)	PhD Thesis, Assiut University
176	Al-Gohary IH	1987	Taxonomic revision of some species of Capparidaceae in Egypt and its verification by some criteria	PhD Thesis, Assiut University
152	Mohamed NH	1980	Studies in the genus Juncus in Egypt	MSc Thesis, Cairo University
135	El-Gazzar A & El_Demerdash MA	1998	Grazing and other ecological pressures in the southern sector of the Gulf of Aqaba	Egyptian Environment Affairs Agency, Cairo
135	Kenawy SKM	2004	Taxonomical and ecological studies on the genus Anabasis growing in Egypt.	MSc Thesis, Al-Azhar University, Cairo
130	Mohamed NH	1986	Flora of Egypt, Systematic Revision of Labiatae	PhD Thesis, Cairo University
130	Mossallam HA et al	2009	Structure of the common plant population along Alamain- Wadi El- Natrun Desert Road	Australian Journal of Basic & Applied Sciences 3
125	Abd El-Ghani MM	1994	Weed plant communities of orchards in Siwa Oasis, Egypt	Feddes Repertorium 105 (5-6), 387- 398
118	Heneidak S	2001	Biosystematic studies on the species of subfamilies Asclepiadoideae and Periplocoideae (family Apocynaceae sensu lato) in Egypt	PhD Thesis, Suez Canal University
117	Boulos L	1966	Flora of the Nile region in Egyptian Nubia.	Feddes Repertorium 73(3): 184-215.
113	El-Hashash SA	1991	Taxonomic and Ecological study of the species of the genus Tamarix L. in Egypt	MSc Thesis, Cairo University
112	Younes AAS	2004	Ecological studies on vegetation of wadi systems in South Sinai, Egypt	PhD Thesis, Suez Canal University
110	FOE6	many	Flora of Egypt (CAIM herbarium?)	6 1-168
108	El-Bous MMM	1995	Taxonomical and chemical studies on the Egyptian species of Melilotus	MSc Thesis, Suez Canal University
108	Shaltout KH et al	2004	Floristic survey of the mountainous region of South Sinai: St. Katherine's Protectorate	UNEP Conservation & Sustainable Use of Medicinal Plants in Egypt, unpublished report
103	Heneidak S et al	2006	Flavonoid glycosides from Egyptian species of the tribe Asclepiadeae (Apocynaceae, subfamily Asclepiadoideae)	Biochemical Systematics & Ecology 34: 575-584
100	El-Wahab RFA	2003	Ecological evaluation of soil quality in South Sinai, Egypt	PhD Thesis, Suez Canal University

99	El-Naggar SMI	1987	Studies on the family Cruciferae in Egypt	MSc Thesis, Assiut University
99	Kamel WM	1986	Taxonomic revision and ecological study of the species of the genus Echinops in Egypt	MSc Thesis, Suez Canal University
85	El-Demerdash MA et al	1996	Distribution of the weed communities in the North East Nile Delta, Egypt	Feddes Repertorium 107(3-4): 219-232
84	Abd El-Ghani MM & Fahmy G	1994	Studies on the threatened woody perennial taxa in the flora of Egypt II. Extinct and endemic taxa	Feddes Repertorium 105 (3-4): 243-250
84	El-Wahab RFA et al	2006	Landforms, vegetation, and soil quality in South Sinai, Egypt	Catrina 1(2): 127-138
84	Moustafa AAR	1986	Ecological and Phytochemical Studies on Some Species of Labiatae Growing in Sinai	MSc Thesis, Suez Canal University
82	Herbarium	many	Herbarium of the Faculty of Science, Alexandria University	
	a further 3105 records from 164 other sources			
